# Draft genome sequence of *Vibrio lentus* VLO8, recovered from the larval culture of the Chilean scallop (*Argopecten purpuratus*)

**DOI:** 10.1128/MRA.00458-23

**Published:** 2023-11-17

**Authors:** Luz Hurtado, Rocío Urtubia, Christopher Concha, Sergio Rodríguez, Juan L. Barja, Rodrigo Rojas

**Affiliations:** 1 Departamento de Acuicultura, Laboratorio de Patobiología Acuática, Universidad Católica del Norte, Coquimbo, Chile; 2 Programa Doctorado en Acuicultura, Facultad de Ciencias del Mar, Universidad Católica del Norte, Coquimbo, Chile; 3 Centro Tecnológico de Innovación Acuícola Aquapacífico, Coquimbo, Chile; 4 Departamento de Microbiología y Parasitología, CIBUS-Instituto de Acuicultura, Universidad de Santiago de Compostela, Santiago de Compostela, Spain; Montana State University, Bozeman, Montana, USA

**Keywords:** *Vibrio lentus*, *Vibrio*, *Argopecten purpuratus*, larvae of molluscs, virulence factors

## Abstract

This announcement reports the genome of *Vibrio lentus* VLO8 recovered from the larval culture of Chilean scallop. The genomes of strain VLO8 have two contigs with a total length of 5,499,980 bp, an average G + C content of 44.22%, a total number of protein-coding genes of 6,439, and 170 RNAs.

## ANNOUNCEMENT

*Vibrio lentus* was described for the first time in oysters from the Mediterranean coast of Valencia (Spain) (1), and it has also been described as a pathogen in octopuses (2) and sea urchins (3). Here, we describe the genome sequence of the potential pathogen *V. lentus* VLO8 isolated from the larval culture of *Argopecten purpuratus*.

The strain VLO8 was isolated on 18 May 2021 from a larval sample of the Chilean scallop collected on 15 May 2021 in the Universidad Católica del Norte Hatchery, Coquimbo, Chile (29.96 S; 71.34 W). The sample was aseptically collected from a sieve with concentrate larvae, then centrifuged at 2,000 × *g* using an Eppendorf centrifuge, and homogenized in 5 mL of sterile seawater. The homogenized obtained was inoculated on Thiosulfate-Citrate-Bile-Salts Sucrose agar (TCBS, BD) prepared with 50% aged micro-filtered (0.45 µm) seawater and Trypticase Soy Agar (BD) supplemented with 2% NaCl (TSA2). Plates were incubated at 20°C for 48 h. The predominant colonies were purified using TSA2 and stored at −85°C in CryoBank vials. The strain selected for genome sequencing was grown on TSA2 agar at 25°C for 24 h prior to use.

The DNA extraction was performed from a pure culture using the DNeasy Blood and Tissue kit (QIAGEN), following the manufacturer’s instructions. The obtained DNA extraction was quantified using a Qubit 3 Fluorometer, and the quality was ensured using a NanoDrop One Spectrophotometer (Thermo Fisher Scientific). The library preparation for the genomic sequencing was based on the Nanopore protocol, Rapid sequencing gDNA-barcoding (SQK-RBK004). The genome sequencing was performed using the Oxford Nanopore MinION, obtaining a total of 50,073 reads. Guppy 6.1.5 included in MinKNOW was used for base calling. Read trimming was performed with the Trim Galore tool implemented in PATRIC. The reads used for assembly after trimming were 28,000 with an average length of 4,633.

The PATRIC server tools were used to assemble the obtained reads through Unicycler v0.4.8 (4) to annotate the resulting genome and determine a taxonomic similarity and phylogenetic affiliation. Mash/MinHash v2.3 (5) and RAxML v8 (6) were used. VLO8 assembly resulted in a 5,499,980 bp length genome, distributed in two contigs corresponding to chromosome 1 (3,492,678 bp) and chromosome 2 (2,007,302 bp), with an average G + C content of 44.22%. The N50 length was 3,492,678 bp. Genome has 6,439 protein-coding sequences (CDS), 130 transfer RNA (tRNA) genes, and 40 ribosomal RNA (rRNA) genes distributed in 2,136 CDS in chromosome 1 and in 1,222 CDS in chromosome 2. Default settings were used for all software unless otherwise indicated.

A BLAST was performed using the 16S ribosomal gene of VLO8 against *Vibrio lentus* 40M4, *Vibrio splendidus DSM19640*, *Vibrio atlanticus CECT223, Vibrio echinoideorum NFHMB010*, and *Vibrio cyclitrophicus FF75*, resulting in a similarity of 99.5%. Therefore, an OrthoANI (7) was performed against these *Vibrio* species, resulting in the highest similarity with *V. lentus* 40M4 (96.66%).

Through the genome analysis with the VFanalyzer web server (8), several complete putative virulence factors were found, including LPS O-antigen, metalloproteinase *hap*/*vvp*, quorum sensing autoinducer-2 and Cholerae autoinducer-1, EPS type II secretion system and the aerolysin AerA in chromosome 1, and a second copy of metalloproteinase *hap*/*vvp* and thermolabile hemolysin *tlh* in chromosome 2.

## Data Availability

The genome was deposited in GenBank under the following accession numbers: WGS JAQAGW000000000, Biosample SAMN32409406, and BioProject PRJNA915966. Raw reads were uploaded to the Sequence Read Archive (SRA) database with SRA accession number SRR26374938.
